# Effects of TCM aromatherapy on post-stroke depression: a meta-analysis of randomized controlled trials

**DOI:** 10.3389/fneur.2025.1623116

**Published:** 2025-08-19

**Authors:** Jing Chen, Yaxuan Yan, Jindan Lv

**Affiliations:** ^1^The Affiliated Traditional Chinese Medicine Hospital, Guangzhou Medical University, Guangzhou, China; ^2^Guangzhou University of Chinese Medicine, Guangzhou, China

**Keywords:** traditional Chinese medicine aromatherapy, post-stroke depression, meta-analysis, therapy, essentail oil

## Abstract

**Background:**

Traditional Chinese Medicine(TCM) aromatherapy has been gradually applied to patients with post-stroke depression (PSD), but uncertainty remains. To systematically evaluate the effectiveness and safety of TCM aromatherapy in the treatment of post-stroke depression, we aim to conclude by synthesizing randomized controlled trials (RCTs).

**Methods:**

We searched the following databases in English and Chinese: China National Knowledge Infrastructure (CNKI), Chinese Biomedical Literature (CBM), Wanfang Database, VIP Database, Chaoxing, PubMed, and The Cochrane Library for randomized controlled trials on the effects of TCM aromatherapy intervention in patients with post-stroke depression. The search period was from the inception of each database to February 20, 2025. After evaluation according to the Cochrane Handbook for Systematic Reviews of Interventions. Meta-analysis was performed using RevMan 5.4.

**Results:**

Ten RCTs involving 614 patients were included. The meta-analysis indicates that, compared to the control group, TCM aromatherapy can ameliorate depressive symptoms in patients with PSD [SMD = 0.53, 95% CI (0.19, 0.87), *p* = 0.003].

**Conclusion:**

The results of this study indicate that TCM aromatherapy alone or combined with other therapies appears to be effective in improving depression symptoms of stroke survivors, but high-quality evidence evaluating TCM aromatherapy for PSD is still needed.

**Systematic reviews registration:**

This study was registered in the International Prospective Register of Systematic Reviews (PROSPERO) database in February 2025. The registration number is CRD42025642863.

## Highlights

- TCM aromatherapy significantly reduces depressive symptoms in patients with PSD compared to conventional therapies.
- TCM aromatherapy reduces anxiety in patients with PSD.
- TCM aromatherapy improves sleep quality in patients with PSD.
- Essential oils like Lavender, SuXexiang, and Rosemary reduce depressive symptoms in patients with PSD.
- TCM aromatherapy regulates emotions through olfactory pathways.

## Background

Post-stroke depression refers to the emotional imbalance and depressive symptoms that occur in patients after a stroke. These symptoms include low mood, self-blame, helplessness, hopelessness, loss of interest and energy, sleep disturbances, changes in appetite, and more (1). Post-stroke depression not only severely impacts the recovery of neurological functions and the quality of life of patients but also increases the risk of stroke recurrence and mortality, placing a heavy burden on patients’ families and society (2). With the intensification of population aging, the incidence of stroke is rising year by year. According to international reports, PSD has a 20 to 60 percent reported prevalence among stroke survivors (3). Although psychological and mental health difficulties after a stroke have been noted for millennia, they have not gotten adequate attention. Instead, greater focus has been placed on motor problems and physical limitations. The current treatment for PSD consists primarily of pharmaceutical and psychosocial therapy. However, pharmaceutical treatments have considerable adverse effects and low compliance, and psychological therapy has problems such as long treatment cycles and expensive expenses (4). Therefore, finding safe, effective, and easily promotable intervention methods for PSD has become a current research hot spot. TCM aromatherapy, as a non-pharmacological intervention, has shown potential in the treatment of psychological and emotional disorders in recent years.

Aromatherapy in traditional Chinese medicine is the application of fragrant herbs in the form of sachets, fumigants, sprays, essential oils, and so on. These are delivered via inhalation, topical application, massage, steaming, or bathing to allow the medicinal compounds to permeate the body, thereby leveraging traditional Chinese herbs’ therapeutic effects for disease treatment and prevention (5). The generally understood mechanism of action consists mostly of activating olfactory receptors in the olfactory bulb. The olfactory bulb then sends signals to the limbic system and hypothalamus, where the brain secretes neurotransmitters like serotonin and dopamine, which further alleviate psychological problems (6). Since it is simple to administer, safe, and convenient, traditional Chinese medicine aromatherapy is widely applied in the treatment of conditions such as insomnia, anxiety, and depression (7). TCM aromatherapy has been applied in the treatment of PSD, and numerous clinical trials have demonstrated its efficacy as a treatment method for PSD (8). However, no high-quality meta-analyses have been conducted to assess the efficacy and safety of TCM aromatherapy in treating PSD. To comprehensively investigate the effectiveness of TCM aromatherapy in treating post-stroke depression, this study examines recently published randomized controlled trials on TCM aromatherapy for post-stroke depression, both domestically and abroad. It aims to provide reliable support for the efficacy of TCM aromatherapy in the treatment of post-stroke depression by incorporating clinical data.

## Literature search

Two researchers (Jing Chen and Yaxuan Yan) searched the China National Knowledge Infrastructure (CNKI), Chinese Biomedical Literature (CBM), Wanfang Database, VIP Database, Chaoxing, PubMed, and The Cochrane Library from their respective inception dates to February 20, 2025. The keywords used are as follows: aromatherapy, fragrance, essential oil, scent therapy, post-stroke depression, post-stroke emotional disorders, depression after stroke, emotional disturbance after stroke. The specific search strategy for PubMed is shown in Table 1 as an example.

**Table 1:** Searching strategy in pubMed.

No	Searching term
1	aromatherapy[Mesh]
2	aromatherapy[Title/Abstract]
3	fragrance[Title/Abstract]
4	essential oil[Title/Abstract]
5	scent therapy[Title/Abstract]
6	aroma therapy[Title/Abstract]
7	1 OR 2 OR 3 OR 4 OR 5 OR 6
8	depression[Mesh]
9	depress[Title/Abstract]
10	emotion[Title/Abstract]
11	psychology[Title/Abstract]
12	disorder[Title/Abstract]
13	8 OR 9 OR 10 OR 11 OR 12
14	stroke[Mesh]
15	Ischemic Stroke[Title/Abstract]
16	Hemorrhagic Stroke[Title/Abstract]
17	Cerebrovascular[Title/Abstract]
18	Brain Vascular Accident[Title/Abstract]
19	Cerebrovascular Stroke[Title/Abstract]
20	Brain Infarction[Mesh]
21	Cerebral Infarction[Mesh]
22	14 OR 15 OR 16 OR 17 OR 18 OR 19 OR 20 OR 21
23	7 and 13 and 22

### Inclusion criteria and exclusion criteria

The inclusion criteria were as follows: (1) Participants: patients included were diagnosed with PSD with no restrictions on diagnostic criteria, gender, age, race, onset time, or source of cases regardless of the cause of the stroke. (2) Interventions: the aromatherapy interventions consisted of inhalation or massage therapy with essential oils bathing, and so on. There were no restrictions on the type of essential oils administered. (3) Comparisons: the control interventions could be placebo, antidepressants, routine care, or other methods. (4) Outcomes: the primary outcome included the HAMD score or other depression scales. (5) Study Design: randomized controlled trials.

The exclusion criteria were as follows:(1) Duplicate publications. (2) Literature for which it is not possible to access the full text and extract valid ending indicators. (3) Literature not in Chinese or English.

### Literature selection and data extraction

Two independent reviewers extracted data from selected RCTs. The essential characteristics such as first author, country, number of treatment and control groups, type of aromatherapy, duration of aromatherapy, type of control intervention, and outcome measures. Any disagreements were reviewed by a third reviewer and resolved by discussion among all reviewers.

### Risk of bias assessment

The methodological quality of the included studies was assessed by two researchers (Jing Chen, and Yaxuan Yan) according to the Cochrane Handbook. Each study was assessed as follows: the risks of selection bias including random sequence generation and allocation concealment, blinding of participants and personnel, blinding of outcome assessment, incomplete outcome data, selective reporting, and other biases. If necessary, we contacted the study authors when relevant information could not be identified from the articles or supplementary materials. In addition, we graded the overall quality of the included trials as high, moderate, or low quality according to previously published high-quality literature. Disagreements were resolved through discussion with a third researcher (Jindan Lv).

### Statistical analysis

Meta-analysis was conducted using RevMan 5.4. In cases where the heterogeneity between studies was *p* > 0.1 and I^2^ < 50%, the fixed-effect model can be chosen. Conversely, if the heterogeneity between studies was *p* ≤ 0.1 and I^2^ ≥ 50%, the random-effect model was used. If the heterogeneity was too large or there was no way to determine the source of heterogeneity, descriptive analysis was used instead.

## Results

### Literature selection

**Figure 1:**
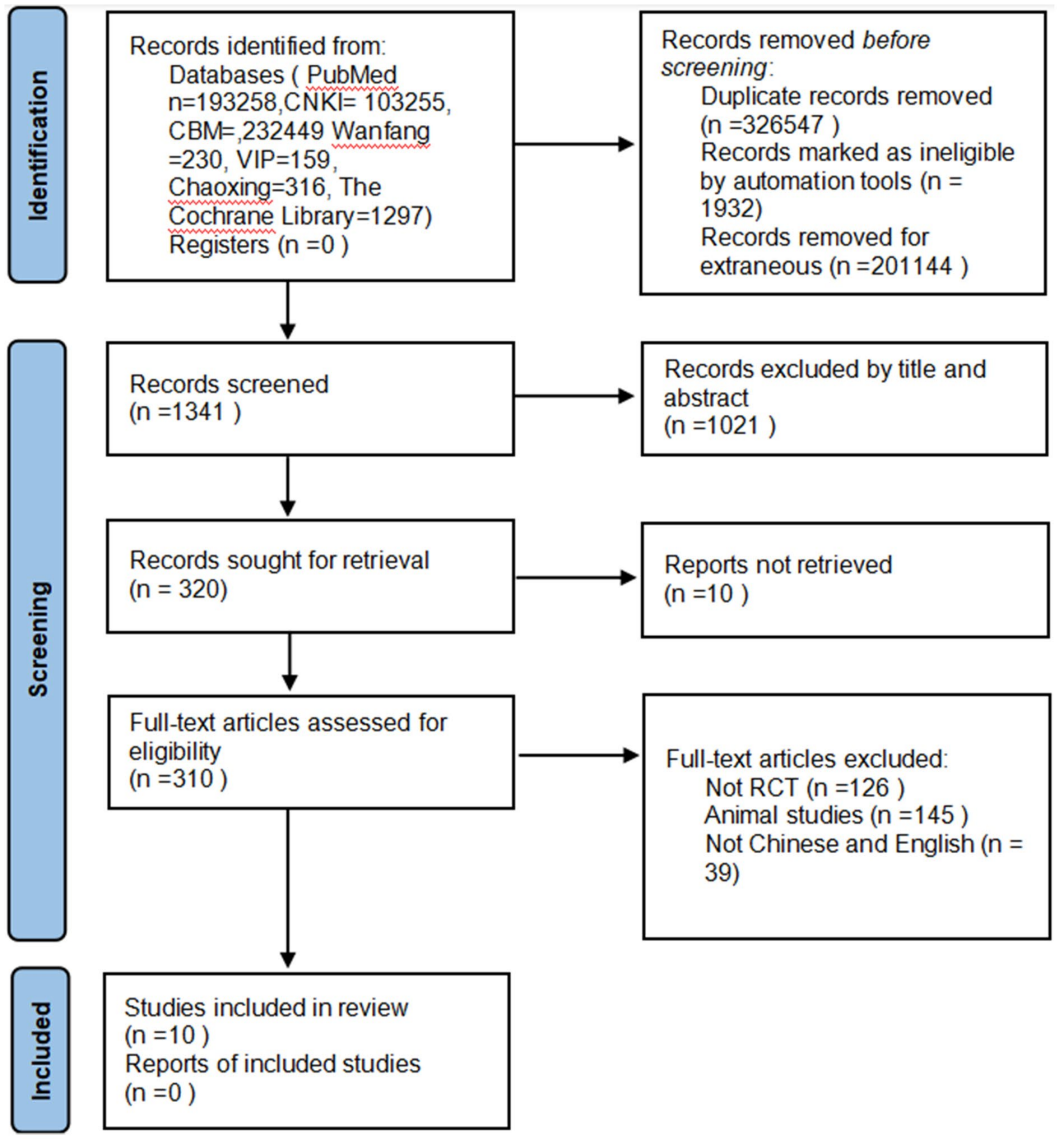
Flow diagram of the literature screening process and results.

The initial search yielded 530,964 relevant articles. After excluding duplicate and irrelevant literature, 1,341 articles remained. Following a preliminary screening by reading titles and abstracts, 320 articles were initially included. Subsequently, after reading the full texts, 310 articles were excluded due to mismatched outcome measures or intervention methods. Upon a second full-text review for further screening, 10 articles were ultimately included in the meta-analysis. The flow chart of the process of study selection is shown in Figure 1.

### Characteristics of the included studies

The characteristics of the included trials are shown in Table 2. All 10 studies were conducted in China, involving a total of 863 PSD patients (9–18). The sample sizes of the included trials ranged from 20 to 50. The treatment duration varied from 4 weeks to 8 weeks, with an average of 6 weeks. 5 trials used aromatherapy alone (11–14, 17), while the rest compared it with other treatments such as auricular point sticking, sertraline, and repetitive transcranial magnetic stimulation in the control groups to explore the effects of combined use of aromatherapy. Table 2 shows the details and characteristics of the included RCTs.

**Table 2:** Characteristics of the included trials.

Study ID	Country	sample		Interventions		Duration	Frequency	Outcomes	Type of intervention
		Treatment	Control	Treatment	Control				
Zhang et al. (16)	China	27	27	Lavender essential oils+rTMS	Conventional therapy+rTMS	4w	40 min/d	HAMD, HAMA	Massage, Inhalation
Chen et al. (9)	China	30	30	Lavender essential oils+Massage	Massage	4w	One night	HAMD-17, NIHSS	Massage, Inhalation
Ma (10)	China	30	30	SuHeXiang essential oil	Distilled water	8w	2 h/d	HAMD-24, PSQI, NIHSS, HAMA	Inhalation
Zhang (11)	China	30	30	Rosemary essential oil	Sertraline	8w	30 min/d	HAMD, HAMA	Inhalation
Wu and Peng (17)	China	30	30	Lavender essential oils	Auricular point sticking	4w	One night	HAMD-17, PSQI	Inhalation
Yin et al. (18)	China	20	20	Lavender essential oils	None	4w	One night	HAMD-17, PSQI	Inhalation
Wang et al. (12)	China	50	50	Chinese herb medicine pillow+sachet	Auricular point sticking	8w	All day	HAMD-24, NIHSS	Inhalation
Tang et al. (13)	China	30	30	Chinese herb medicine pillow+Auricular point sticking	Auricular point sticking	4w	One night	HAMD-17, NIHSS	Inhalation
Yu et al. (15)	China	30	30	Lavender essential oils	Conventional therapy	30d	6-8 h/d	HAMD-21, PSQI	Inhalation
Luo (14)	China	30	30	Compound essential oil	Almond oil	4w	30-35 min/d	HAMD-24, NIHSS, PSQI, HAMA	Massage

w = weeks, d = days, h = hours, HAMD = Hamilton Depression Scale, HAMA = Hamilton Anxiety Scale, NIHSS = National Institute of Health stroke scale, PSQI = Pittsburgh Sleep Quality Index.

### Risk of bias assessment

**Figure 2:**
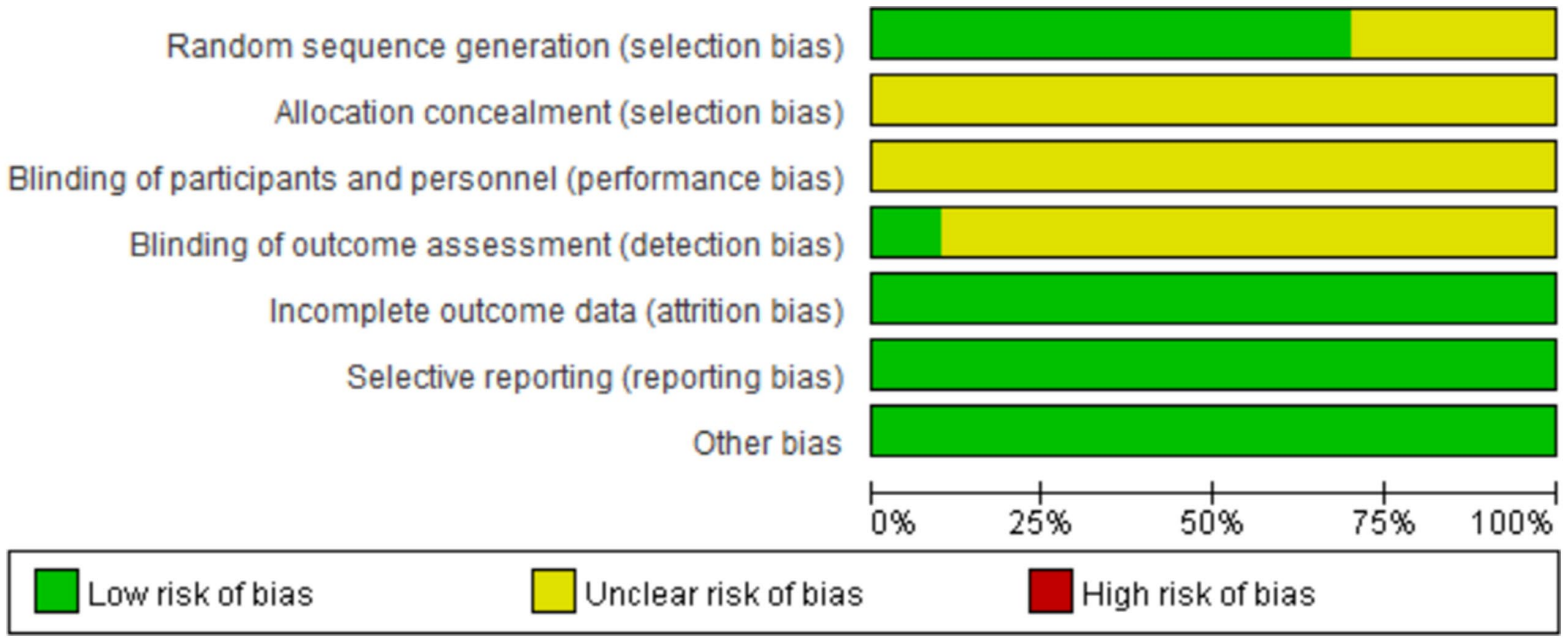
Risk of bias for included studies.

All the literature mentioned randomized grouping, among which 6 articles used the random number table method for grouping (10–12, 15, 16, 18). 1 article used convenience sampling (17), and the remaining 3 articles only mentioned “randomization (16–18).” One article explicitly stated that the study adopted a single-blind trial (9, 13, 14), while the others did not mention the concealment of allocation methods or the implementation of blinding. There was one study with drop-out cases, but it did not affect the overall results. The research data were complete, all literature was reported as originally planned, baseline data were comparable, and there were no other biases. The quality assessment is shown in Figure 2.

### Study outcomes

#### Effects of TCM aromatherapy on depression

**Figure 3:**
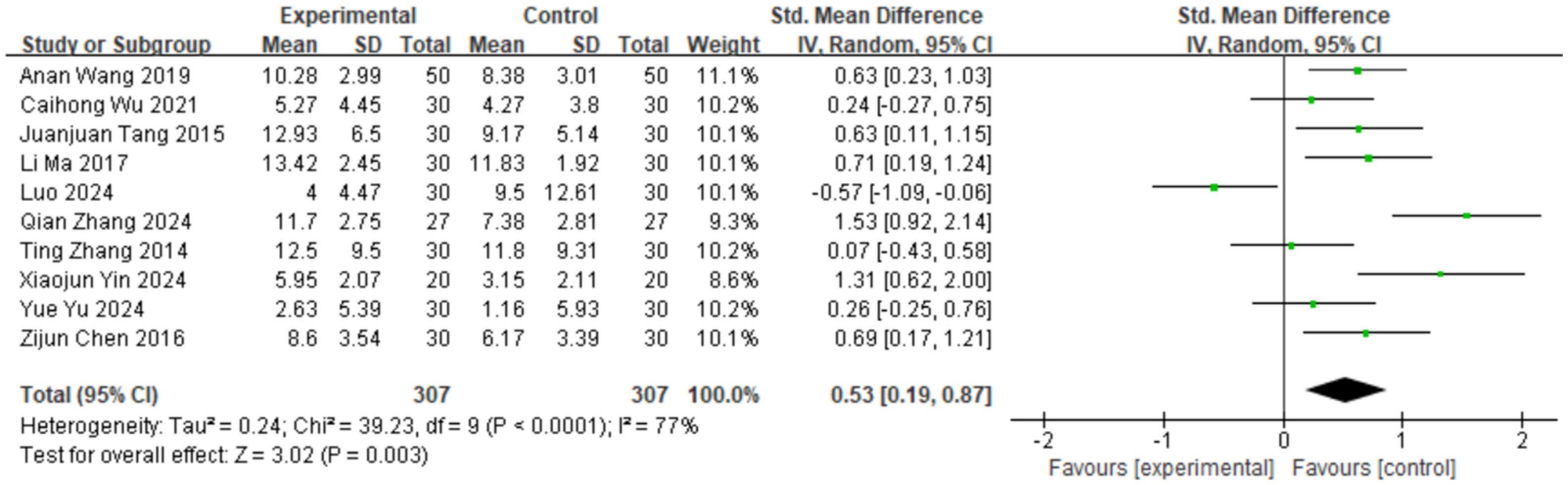
Forest plot for the HAMD scale.

Among the 10 included studies, 10 utilized the HAMD as a research metric (9–18), while the versions of the scales used are all different. Therefore, the standardized mean difference was chosen for representation. As shown in Figure 3, the heterogeneity test in this study indicated significant heterogeneity among the studies (*I*^2^ = 77%, *p* < 0.0001), leading to the adoption of a random-effects model. The results showed that the depression scale scores in the aromatherapy group were lower than those in the control group, with a statistically significant difference between the two groups [SMD = 0.53, 95% CI (0.19, 0.87), *p* = 0.003].

#### Effects of TCM aromatherapy on sleep

**Figure 4:**
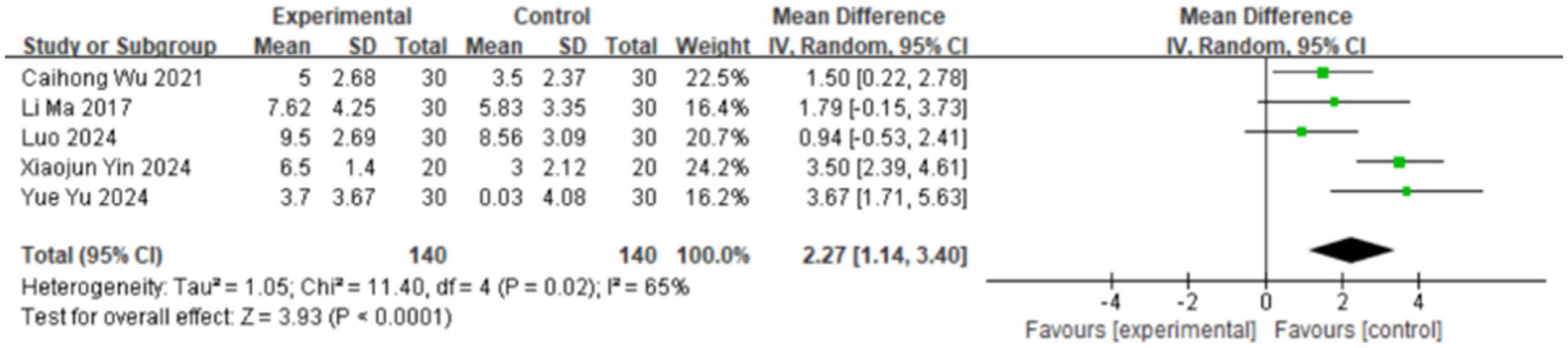
Forest plot for the PSQI scale.

Five studies measured the sleep conditions of the research subjects (11, 13, 14, 17, 18), all using the Pittsburgh Sleep Quality Index, involving 280 cases. As shown in Figure 4, there was significant heterogeneity among the studies (*I*^2^ = 65%), so a random-effects model was used for the meta-analysis. The meta-analysis results showed that TCM aromatherapy is beneficial in improving the sleep quality of patients with post-stroke depression [MD = 2.27, 95% CI (1.14 3.40), *p* < 0.0001].

#### Effects of TCM aromatherapy on anxiety

**Figure 5:**
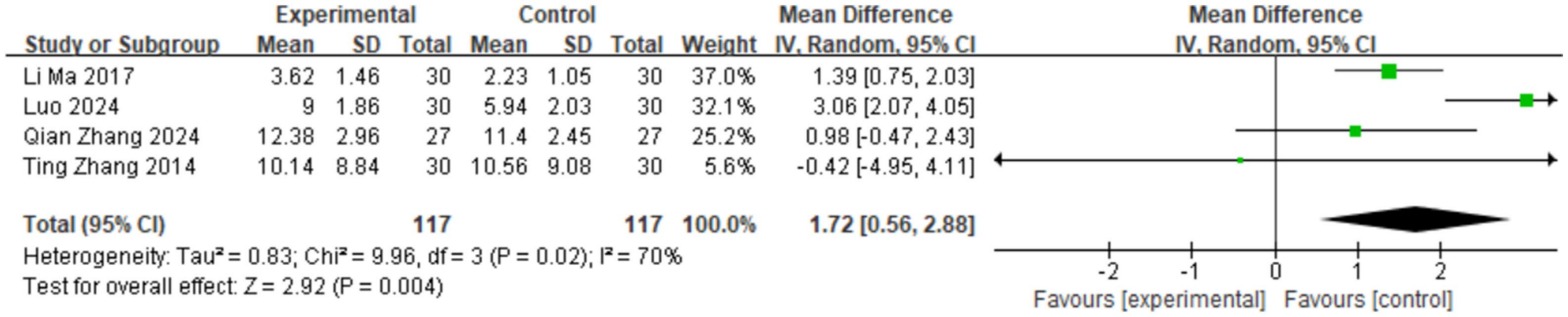
Forest plot for the HAMA scale.

Four studies measured the anxiety levels of the research subjects (9, 11, 12, 18), all using the Hamilton Anxiety Scale, involving 234 cases. As shown in Figure 5, the heterogeneity among the studies was high (*I*^2^ = 70%), so a random-effects model was used for the Meta-analysis. The meta-analysis results showed that TCM aromatherapy is beneficial in alleviating anxiety in patients with post-stroke depression [MD = 1.72, 95% CI (0.56, 2.88), *p* = 0.004].

### Subgroup analysis

**Figure 6:**
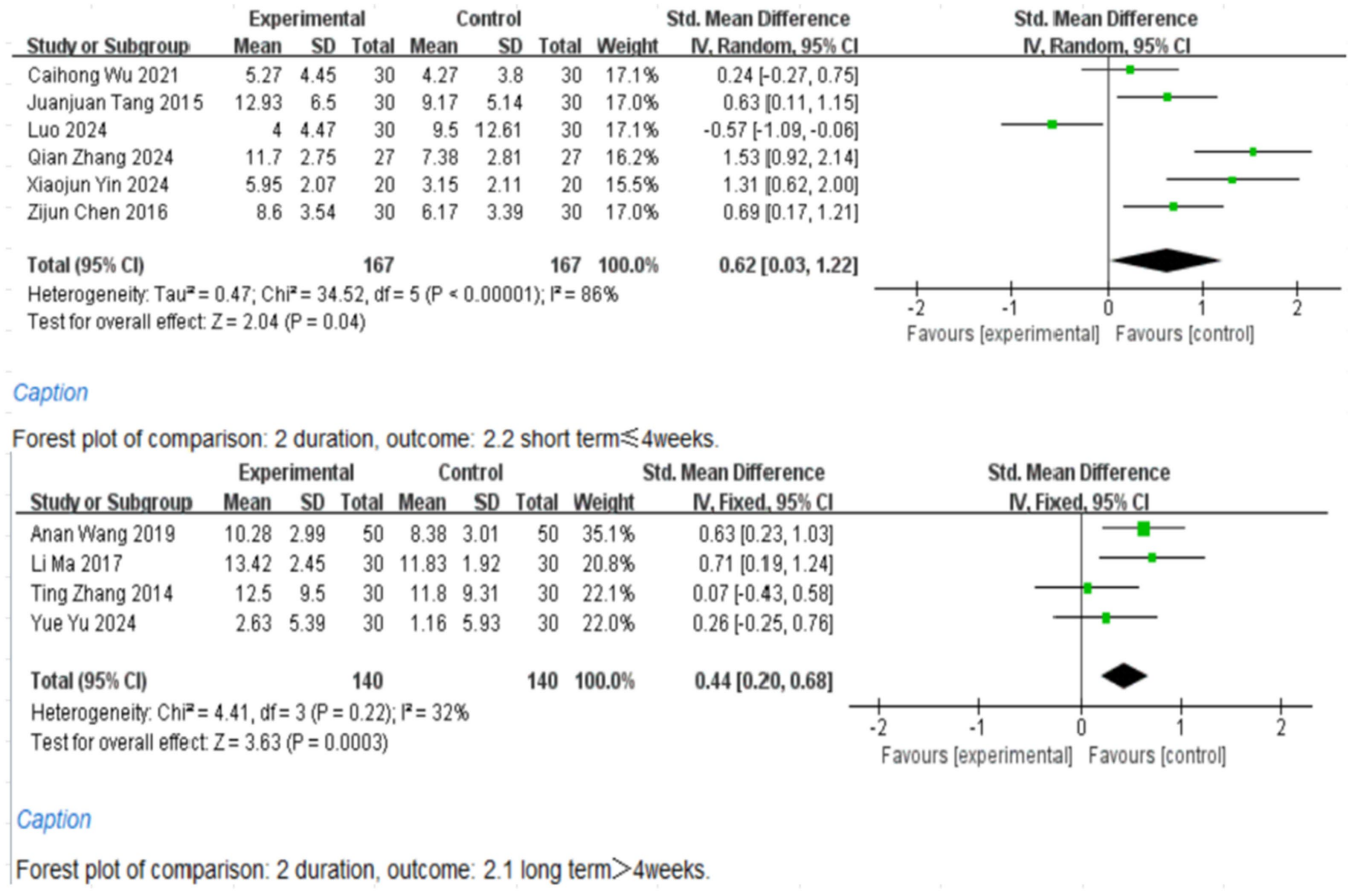
Subgroup analysis according to the duration of aromatherapy intervention.

**Figure 7:**
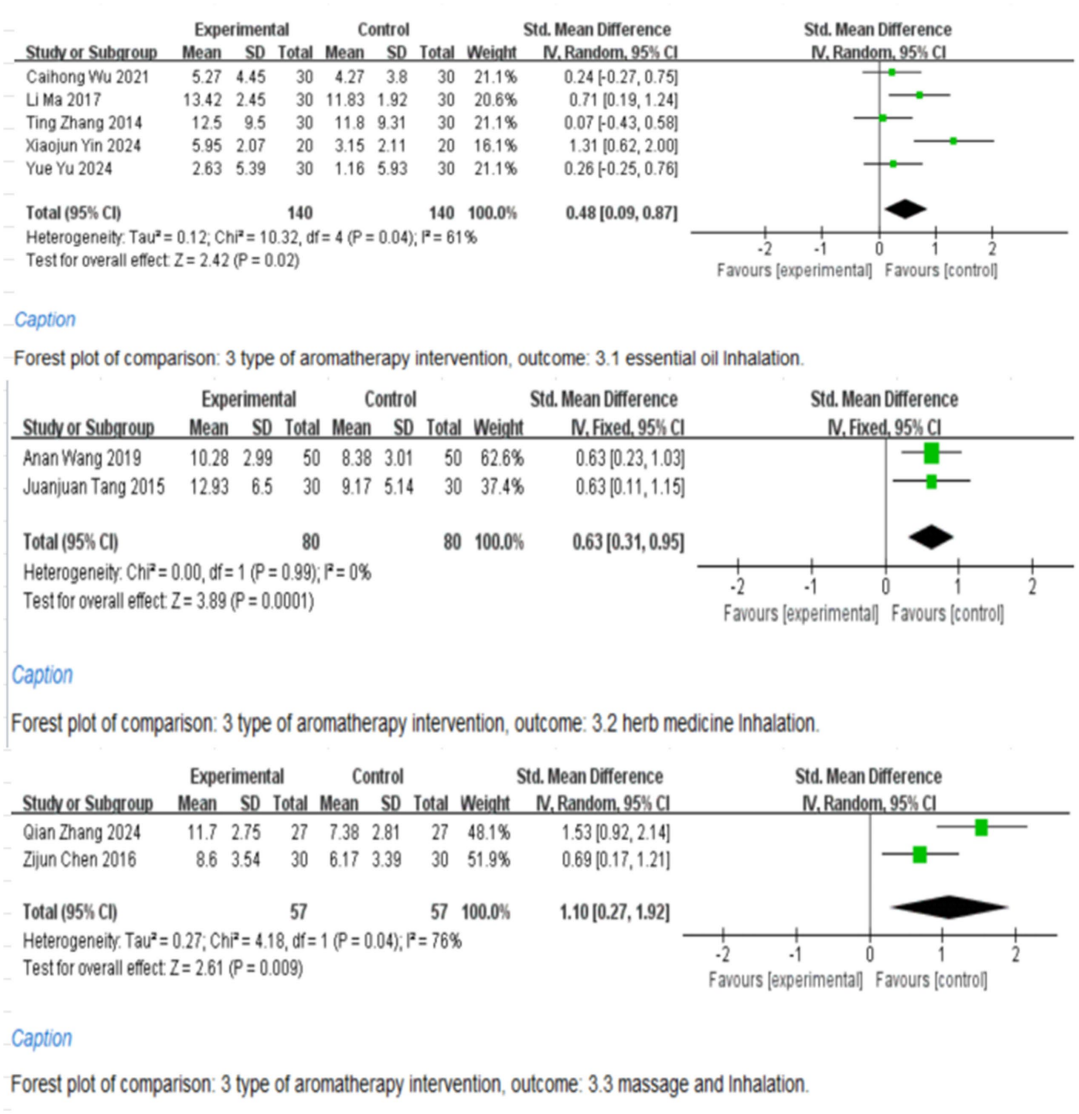
Subgroup analysis according to the type of aromatherapy intervention.

The studies were grouped based on the duration of aromatherapy intervention (≤4 weeks, >4 weeks), with 6 studies intervening for 4 weeks (9, 10, 13, 14, 16, 18), 3 studies for 8 weeks (11, 12, 15), and 1 study for 30 days (17) (Figure 6). Subgroup analysis was conducted according to the type of aromatherapy, categorized into essential oil inhalation, traditional Chinese medicine inhalation, and essential oil inhalation combined with essential oil massage. Among them, 2 studies adopted inhalation combined with massage, 2 studies adopted traditional Chinese medicine inhalation, and 5 studies used inhalation alone (Figure 7). The results showed that TCM aromatherapy interventions in each subgroup effectively alleviated depressive symptoms in patients with PSD. However, the heterogeneity among subgroups did not completely disappear, indicating that the aforementioned factors were not entirely the source of heterogeneity.

### Publication bias and sensitivity analysis

Sensitivity analyses of depressive symptoms in PSD patients treated with TCM aromatherapy were performed, and the heterogeneity was not significantly altered after excluding each study one by one. Due to the limited inclusion of literature, a funnel plot could not be drawn to determine publication bias.

## Discussion

This meta-analysis, involving 10 studies, demonstrates that TCM aromatherapy alone or in combination with other interventions seemed to be more effective in improving depression symptoms of PSD. Although PSD is known to have a detrimental effect on functional recovery and quality of life after stroke, it is often overlooked and untreated (19). Currently, the main treatments for post-stroke depression are antidepressants and non-pharmacological therapy. However, current treatments have many limitations, such as expensive treatment and significant adverse effects (20). TCM aromatherapy is a simple, effective, and economical alternative intervention method, which has obvious effects on alleviating depressive symptoms after stroke. At the present time, the commonly used essential oils in treating PSD patients include lavender, sweet orange, rose, and bergamot. Research indicates that lavender has stress-relieving and antidepressant properties (21). The majority of the material in this study makes use of both massage and inhalation. Through inhalation, the different kinds of plant essential oils used in aromatherapy enter the nasal cavity, stimulating the nose’s olfactory cells. From there, they travel via the olfactory pathway to the limbic system and hypothalamus, where the brain releases neurotransmitters like dopamine and serotonin that can help reduce depressive symptoms (22). It is absorbed by the entire body through the bloodstream after being massaged into the skin’s pores. From there, it travels to the brain, where it encourages the release of neurochemicals that have a calming, relaxing effect (23). Additionally, massage and essential oils promote immunological function by increasing lymphocyte counts, reducing fatigue, and alleviating depressed symptoms (24). Furthermore, research progress of essential oil had provided scientific basis for an essential oil to be a new choice for relieving depression and treating depression (25). Animal studies have also shown that Lavender essential oil ameliorates depression-like behavior and increases neurogenesis and dendritic complexity in rats (26). The majority of the included studies had brief intervention periods, despite the fact that our meta-analysis demonstrates that traditional Chinese aromatherapy can successfully alleviate depressive moods in PSD patients in the short term. Although subgroup analysis by intervention length showed few differences in improved effects, more research is still needed to determine the long-term efficacy. TCM aromatherapy is still in its infancy in China, while being comparatively well-established overseas. Due to the lack of established criteria, the selection of essential oils in therapeutic practice is now primarily reliant on personal experience, which could produce inconsistent outcomes among research. Therefore, further research is needed to explore the effects of different types of aromatic agents and their application methods for PSD patients, aiming to establish a unified and rigorous evaluation standard and provide reliable evidence for improving patient comfort.

### Limitations

Limitations of this study: (1) Evaluation metrics: Among the 10 included randomized controlled trials, the assessment indicators for depression were not consistent. Current research primarily focuses on scale scores, with only a few incorporating physiological indicators. It is recommended that future researchers adopt more objective metrics to ensure the objectivity and authenticity of the studies. (2) Study design: Most of the included studies had short intervention durations and did not involve long-term follow-up after the intervention. Therefore, the long-term effects of traditional Chinese aromatherapy on depression remain unclear and warrant further in-depth research. (3) Sample size: The sample sizes in the included studies were relatively small. Future studies could increase the sample size to enhance the generalizability of the conclusions. (4) Literature quality: The majority of the included studies had suboptimal use of randomization and blinding methods, resulting in lower quality ratings. The assessment methods and intervention measures in the included literature were not entirely consistent, which may affect the stability of the findings.

In summary, the results of this meta-analysis suggest that TCM aromatherapy has a certain improvement effect on depressive symptoms in patients with post-stroke depression. However, due to the generally low quality of the included literature, higher-level and higher-quality studies are needed to confirm the accuracy and reliability of the results. Additionally, larger sample sizes and longer-term studies are required to validate further the effectiveness of TCM aromatherapy in alleviating depressive symptoms in PSD patients, thereby providing more reliable evidence for improving patient comfort and quality of life.

## Conclusion

This review strengthens the evidence that TCM aromatherapy could reduce the degree of PSD. However, there is still a lack of moderate and high-quality evidence to support this conclusion. Further methodologically rigorous and adequately powered primary studies are necessary to assess the effect of TCM aromatherapy on PSD.

## Data Availability

The original contributions presented in the study are included in the article/supplementary material, further inquiries can be directed to the corresponding author/s.
